# IL-17-producing γδ T cells enhance bone regeneration

**DOI:** 10.1038/ncomms10928

**Published:** 2016-03-11

**Authors:** Takehito Ono, Kazuo Okamoto, Tomoki Nakashima, Takeshi Nitta, Shohei Hori, Yoichiro Iwakura, Hiroshi Takayanagi

**Affiliations:** 1 Department of Immunology, Graduate School of Medicine and Faculty of Medicine, The University of Tokyo, Hongo 7-3-1, Bunkyo-ku, Tokyo 113-0033, Japan; 2 Japan Science and Technology Agency (JST), Exploratory Research for Advanced Technology (ERATO) Program, Takayanagi Osteonetwork Project, Hongo 7-3-1, Bunkyo-ku, Tokyo 113-0033, Japan; 3 Department of Cell Signaling, Graduate School of Medical and Dental Sciences, Tokyo Medical and Dental University, Yushima 1-5-45, Bunkyo-ku, Tokyo 113-8549, Japan; 4 Precursory Research for Embryonic Science and Technology (PRESTO), Japan Science and Technology Agency (JST), Yushima 1-5-45, Bunkyo-ku, Tokyo 113-8549, Japan; 5 Japan Agency for Medical Research and Development, Core Research for Evolutional Science and Technology (AMED-CREST), Yushima 1-5-45, Bunkyo-ku, Tokyo 113-8549, Japan; 6 Laboratory for Immune Homeostasis, RIKEN Center for Integrative Medical Science, Suehiro-cho 1-7-22, Tsurumi-ku, Yokohama, Kanagawa 230-0045, Japan; 7 Research Institute for Biological Sciences, Tokyo University of Science, Yamazaki 2669, Noda, Chiba 278-0022, Japan

## Abstract

Immune responses are crucial not only for host defence against pathogens but also for tissue maintenance and repair after injury. Lymphocytes are involved in the healing process after tissue injury, including bone fracture and muscle damage. However, the specific immune cell subsets and mediators of healing are not entirely clear. Here we show that γδ T cells produce IL-17A, which promotes bone formation and facilitates bone fracture healing. Repair is impaired in IL-17A-deficient mice due to a defect in osteoblastic bone formation. IL-17A accelerates bone formation by stimulating the proliferation and osteoblastic differentiation of mesenchymal progenitor cells. This study identifies a novel role for IL-17-producing γδ T cells in skeletal tissue regeneration.

## Abstract (web summary)

γδ T cells are innate-like lymphocytes that regulate immune responses by producing IL-17A or IFN-γ, but have no known role in bone healing. Here the authors show a nonimmune bone-regenerative function of IL-17A produced by the Vγ6+ subset in mice.

## Graphical abstract

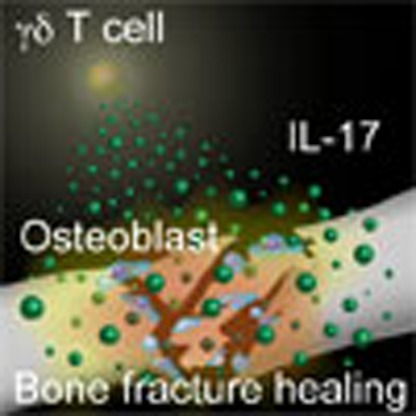

The immune system protects the body by eradicating pathogenic microorganisms but has alternative physiological and pathological roles in a variety of biological systems including the musculoskeletal system. T cells are important in the pathogenesis of erosive arthritis and spondyloarthropathy accompanied with enhanced bone formation^1^,^2^,^3^. In addition, inflammatory reactions are involved in ectopic bone formation in fibrodysplasia ossificans progressiva^4^ and one report indicates that regulatory T cells are crucial for muscle repair^5^. Bone repair after injury is also associated with immune reactions.

Fractured bone regenerates through a cascade of events: haematoma formation, inflammation, callus formation and bone remodelling^6^. On bone fracture, blood vessels near the injury site rupture, resulting in the formation of a haematoma. The haematoma is infiltrated by immune cells, including neutrophils, macrophages and lymphocytes, which induce acute inflammation, as well as the removal of dead cells and tissue debris. After the resolution of inflammation, mesenchymal progenitor cells accumulate around the fractured site to form granulation tissue. Neovascularization in the injury site is also observed^6^. The mesenchymal progenitor cells differentiate into chondrocytes and osteoblasts, to undergo endochondral and intramembranous ossification, forming callus that bridges bone fragments. The callus is replaced by mature bone tissue by bone remodelling in the later stage of repair so that the fractured bone restores its original shape and function.

As T cells are present in the haematoma and mice deficient in lymphocytes reportedly exhibit delayed or accelerated bone fracture healing^7^,^8^, it is suggested that T cells contain multiple subsets with different functions in bone repair. Effector memory CD8^+^ T cells have been reported to delay fracture healing^9^. However, the specific T-cell subsets that promote healing and the mediating factors involved remain to be elucidated.

γδ T cells are innate-like lymphocytes that are distributed preferentially to peripheral tissues and can exert tissue-regenerative functions^10^,^11^. Here we show that interleukin (IL)-17A is highly induced immediately after bone injury and promotes bone regeneration by accelerating osteogenesis via its effects on injury-associated mesenchymal cells. Furthermore, we reveal that Vγ6^+^ γδ T cells (T cell receptor (TCR) nomenclature of Heilig and Tonegawa)^12^ proliferate in the injury site and function as the crucial producer of IL-17A in fracture healing.

## Results

### IL-17A is induced in the repair tissue after bone injury

To determine which type of T cells are involved in the bone regeneration that occurs after injury, we analysed the messenger RNA (mRNA) expression of the T-cell-related cytokines in the bone regeneration process following the introduction of a femoral cortical bone defect by drill-hole injury (Supplementary Fig. 1a)^13^. This model essentially recapitulates the intramembranous bone formation process, enabling a simplified quantification of repaired bone and the preservation of the bone marrow as the result of not using a fixation system. After bone injury, massive proliferation of fibroblastic cells in the drill hole, along with an infiltration of inflammatory cells and vascularization occurred (Supplementary Fig. 1b–d). Regenerating skeletal muscle cell layer (Supplementary Fig. 1c) and a thickening periosteum (Supplementary Fig. 1d) were observed extending into the proliferating fibroblastic cell layer. As the regenerative tissue in the drill hole was continuous with the one around the bone, we harvested the cells from the tissues in the drill hole, periosteum and injured skeletal muscle, and defined them as the cells of the repair tissue.

**Figure 1:**
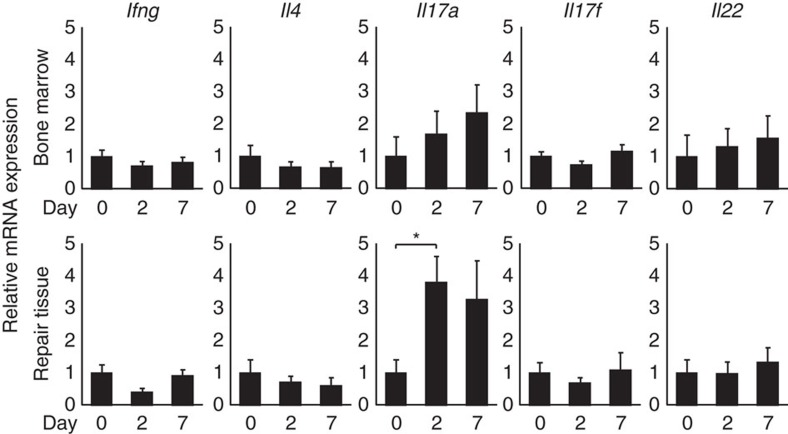
IL-17A is induced in the repair tissue after bone injury. mRNA expression levels of T-cell-related cytokines in the cells of the bone marrow and the repair tissue of wild-type mice 2 and 7 days after injury compared with non-treated (day 0) tissue (*n*=3−6 per time point). Statistical analysis was carried out using one-way ANOVA with Dunnett's test. Error bars denote the mean±s.e.m. *P<0.05.

There was no significant increase in the expression of *Ifng*, *Il4*, *Il17f* or *Il22* in the bone marrow or the repair tissue; however, the expression of *Il17a* in the repair tissue, not in the bone marrow, was significantly increased 2 days after injury (Fig. 1 and Supplementary Fig. 1e). These results suggest that IL-17A plays a role in the process of bone regeneration after injury.

### IL-17A promotes bone regeneration after injury

**Figure 2:**
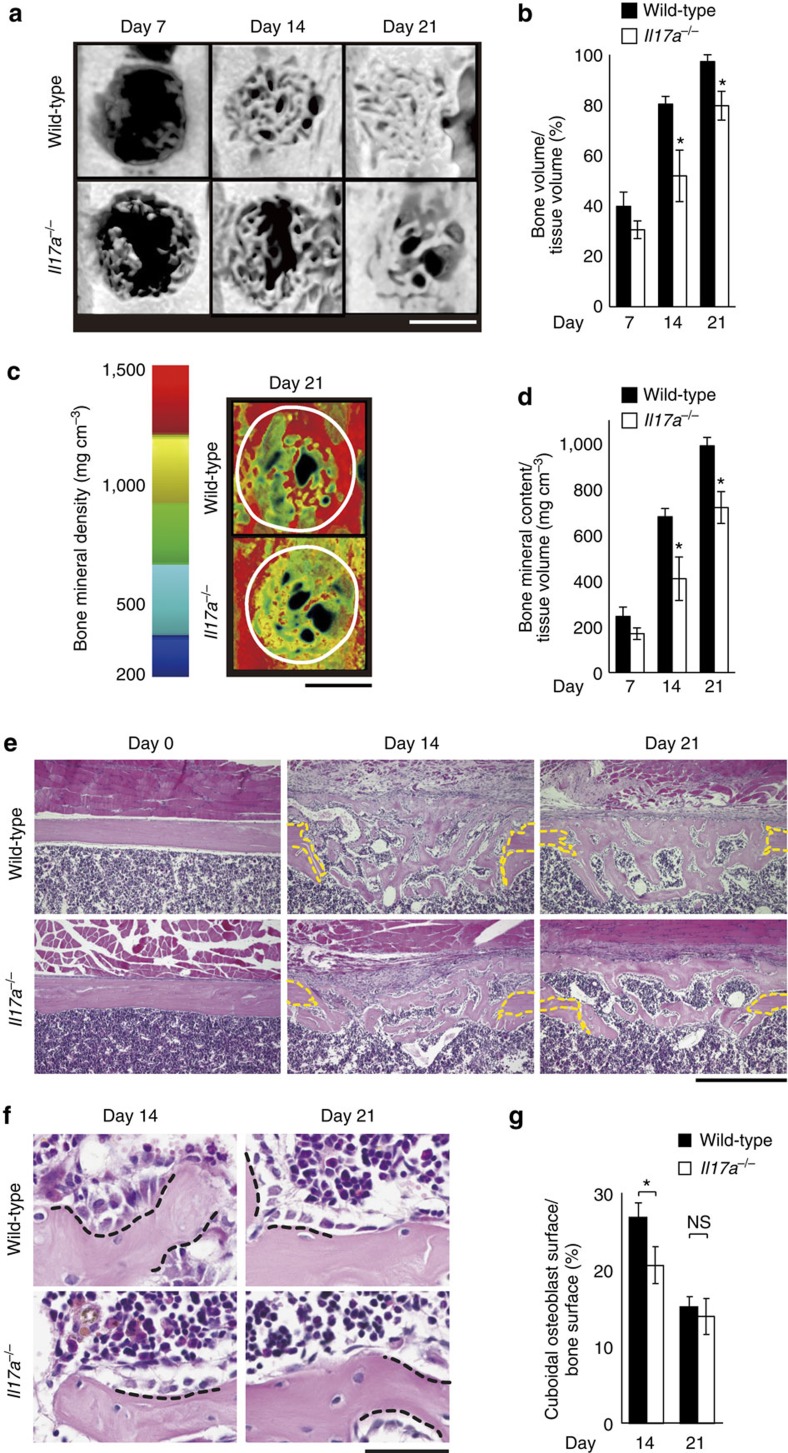
IL-17A promotes bone regeneration after injury by enhancing osteoblast function. (**a**) Micro-CT images of the drill holes in the wild-type and *Il17a*^−/−^ mice after injury. Scale bar, 500 μm. (**b**) Quantification of bone formation in the drill holes (*n*=4−8 per time point per genotype). (**c**) Visualization of the bone mineral density of the newly formed bone in the drill holes. Images were constructed by colouring the micro-CT images according to the CT values. The lines denote the original shapes of the drill holes. Scale bar, 500 μm. (**d**) Bone mineral content in the drill holes (*n*=4−8 per time point per genotype). (**e**) Histological images of the femur of the wild-type and *Il17a*^−/−^ mice stained with HE staining. The lines indicate the edge of the drill hole. Scale bar, 500 μm. (**f**) Bone surface lined with cuboidal osteoblasts (dotted lines) on the newly formed bone. Scale bar, 50 μm. (**g**) Cuboidal osteoblast surface per bone surface. The bone surface covered with bone-forming osteoblasts was quantified by bone histomorphometric analysis (*n*=3 per time point per genotype, 6 representative sections per each femur). Statistical analysis was carried out using Student's *t* test for each time point in Fig. 2. Error bars denote the mean±s.e.m. *P<0.05; NS, not significant.

To determine the role of IL-17A in the regenerative process after injury, we assessed bone regeneration using *Il17a*^−/−^ mice by measuring the level of closure of the drill hole with micro-computed tomography (CT). We found that 14 and 21 days after injury, the volume of the newly formed bone tissue in the drill hole was smaller in the *Il17a*^−/−^ mice than wild-type mice (Fig. 2a,b). The bone mineral content within the drill hole was lower in the *Il17a*^−/−^ mice than in the wild-type mice (Fig. 2c,d). Although it has been suggested that IL-17F is involved in fracture repair^8^, there was no significant difference in bone regeneration in the wild-type and *Il17f*^−/−^ mice (Supplementary Fig. 1f–i).

We then evaluated bone formation by osteoblasts and bone resorption by osteoclasts at the injury site using histomorphometry. New bone was formed as trabecula-like structures in the drill hole in both the wild-type and *Il17a*^−/−^ mice (Fig. 2e). To investigate the number of bone-forming osteoblasts on the bone surface, we measured the ratio of the bone surface covered by cuboidal osteoblasts, which are known to have a high capacity for synthesizing bone matrix. The ratio of the cuboidal osteoblast surface to the bone surface was lower in the *Il17a*^−/−^ mice than in the wild-type mice (Fig. 2f,g). There was no significant difference in the ratio of the eroded surface and the number of osteoclasts between the wild-type and *Il17a*^−/−^ mice during the process of bone regeneration (Supplementary Fig. 2a–c). These results suggest that IL-17A promotes bone regeneration by activating osteoblastic bone formation without affecting osteoclastic bone resorption.

### Injury tissue contains IL-17A-responsive PαS cells

The differentiation of mesenchymal cells is regulated by various cytokines. Among these cytokines, IL-6 and tumour necrosis factor, which are induced by IL-17A, are known to have the capacity to promote bone fracture healing^6^. However, there was no significant difference in the expression of *Il6* and *Tnfa* in the repair tissues of wild-type and *Il17a*^−/−^ mice (Supplementary Fig. 3a), indicating that IL-17A promotes bone formation independently of these cytokines.

**Figure 3:**
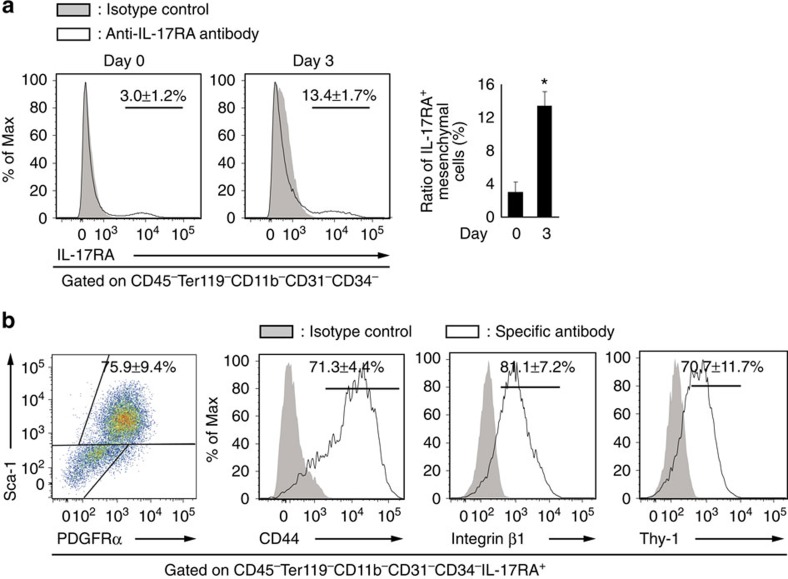
Mesenchymal cells in the injury site contain IL-17A-responsive PαS cells. (**a**) The ratio of IL-17RA^+^ cells in CD45^−^Ter119^−^CD11b^−^CD31^−^CD34^−^ mesenchymal cells in the repair tissues of non-treated and injured mice (*n*=6 per time point). (**b**) The expression of MSC markers on IL-17RA^+^ mesenchymal cells of the repair tissue (*n*=3−6). Most of the IL-17RA^+^ mesenchymal cells were PαS cells positive for both platelet-derived growth factor receptor-α (PDGFRα) and Sca-1. Statistical analysis was carried out using Student's *t*-test. Error bars denote the mean±s.e.m. *P<0.05.

We analysed the expression of the IL-17A receptor IL-17RA on the cells of the repair tissue after the haematopoietic cell population and endothelial cell population positive for CD45, Ter119, CD11b, CD31 or CD34 had been gated away. IL-17RA was expressed on the mesenchymal cells in the repair tissue and the ratio of IL-17RA-expressing mesenchymal cells was significantly increased after the injury (Fig. 3a). Most of the IL-17RA^+^ mesenchymal cells in the injury tissue were positively stained for both platelet-derived growth factor receptor (PDGFR)-α and Sca-1 (Fig. 3b). PDGFRα^+^Sca-1^+^ cells (PαS cells) are thought to be an enriched population of mesenchymal stem cells (MSCs)^14^. Mesenchymal cells in the repair tissue were highly positive for other MSC markers: CD44, Integrin β1 and Thy-1 (Fig. 3b). We further confirmed that IL-17RA^+^ mesenchymal cells and PαS cells were actually present in the drill hole even when we removed the outer layer of the repair tissue (Supplementary Fig. 3b).

### IL-17A enhances osteoblastogenesis *in vitro*

**Figure 4:**
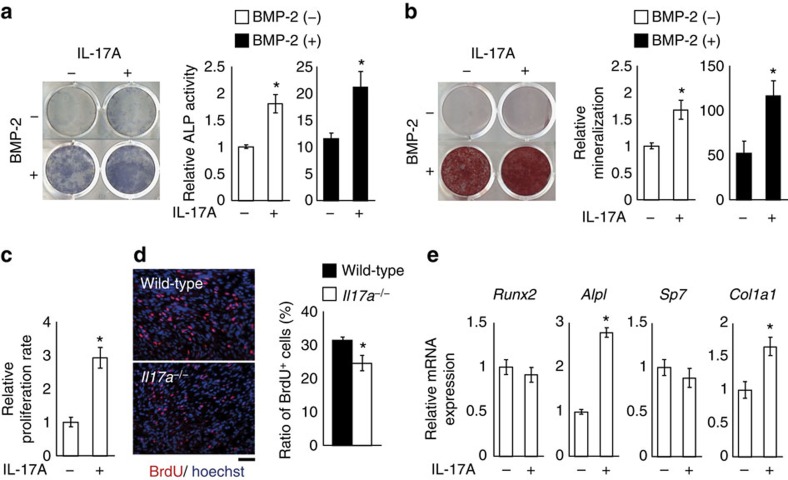
IL-17A enhances bone formation by injury-associated mesenchymal cells. (**a**) The effect of IL-17A on ALP activity of injury-associated mesenchymal cells in the presence or absence of BMP-2. (**b**) The effect of IL-17A on *in vitro* mineralization as analysed by Alizarin Red S staining and quantification. (**c**) *In vitro* proliferation of injury-associated mesenchymal cells quantified by BrdU incorporation assay. (**d**) Proliferation of the cells within the drill hole of the wild-type and *Il17a*^−/−^ mice as quantified by *in vivo* BrdU incorporation assay (*n*=3 per genotype). BrdU^+^ cells were detected by immunofluorescence and the ratio of BrdU^+^ cells was calculated. Scale bar, 50 μm. (**e**) The mRNA expression levels of osteoblast genes in the injury-associated mesenchymal cells. All the data of the *in vitro* experiments were obtained from three independent experiments with triplicate wells. Statistical analysis was carried out using Student's *t*-test throughout Fig. 4. Error bars denote the mean±s.e.m. *P<0.05.

The above results led us to examine the effects of IL-17A on injury-associated mesenchymal cells. The activity of alkaline phosphatase (ALP), an osteoblast differentiation marker, and mineralization were upregulated by IL-17A in both the presence and absence of bone morphogenetic protein (BMP)-2 (Fig. 4a,b). IL-17A decreased mineralization and bone nodule formation in murine calvarial cells (Supplementary Fig. 4a–c) consistent with a previous report^15^, suggesting that IL-17A exerts distinct effects on bone formation depending on the cellular origin.

How does IL-17A regulate osteoblastic bone formation? 5-Bromodeoxyuridine (BrdU) incorporation assay showed that IL-17A significantly increased the proliferation of the injury-associated mesenchymal cells (Fig. 4c). Consistent with this, immunohistological analysis showed that the number of BrdU^+^ cells within the drill hole in *Il17a*^−/−^ mice was significantly lower than that in the wild-type mice (Fig. 4d). We analysed the effect of IL-17A on the mRNA expression of osteoblast genes. The expression of *Runx2* and *Sp7*, the essential transcription factors for osteoblastogenesis, was unchanged, whereas the mRNA of certain osteoblast genes including *Alpl* and *Col1a1* was upregulated (Fig. 4e). Thus, IL-17A promotes bone formation in injury-associated mesenchymal cells through the stimulation of both osteoblast proliferation and differentiation. To examine the contribution of soluble factors released from injury-associated mesenchymal cells, we performed a comprehensive mRNA expression analysis on PαS cells stimulated with IL-17A and/or BMP-2. Among the soluble factors and their receptors related to osteoblast differentiation, *Fgf2*, *Pdgfa*, *Pdgfc* and *Tgfb1* were found to be upregulated by these cytokines, suggesting that IL-17A may indirectly promote osteoblastogenesis via these factors at least in part (Supplementary Fig. 4d).

### Vγ6^+^ γδ T cells produce IL-17A on bone injury

**Figure 5:**
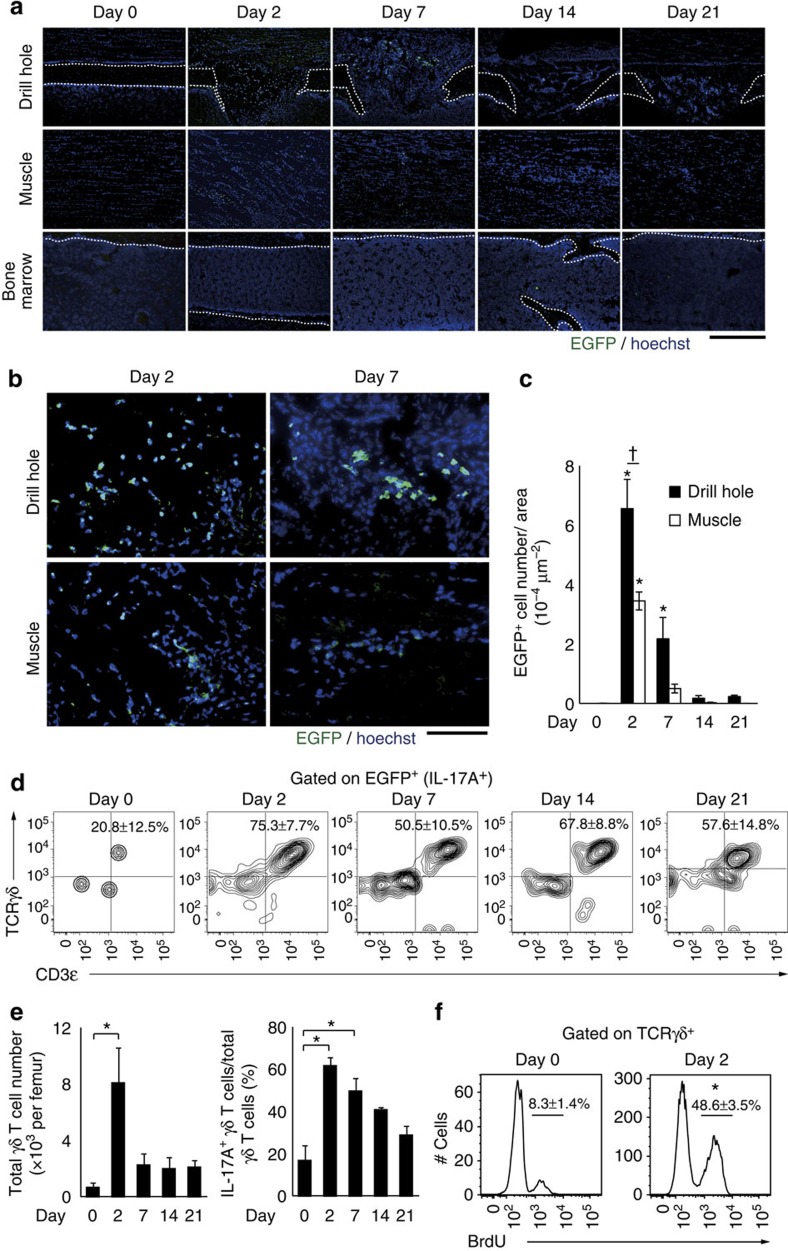
γδT cells produce IL-17A on bone injury. (**a**) IL-17A-producing cells in the drill hole, skeletal muscle and bone marrow. *Il17a*^*gfp*/*gfp*^ mice were used for the detection of IL-17A-producing cells as EGFP^+^ cells. Nuclei were stained by Hoechst33342. Scale bar, 500 μm. (**b**) High-magnification images of the drill hole and the injured skeletal muscle 2 and 7 days after injury. Scale bar, 50 μm. (**c**) Quantification of IL-17A-producing cells within the drill hole and the injured muscle (*n*=3 per time point, 2 representative sections per each femur). Statistical analysis was carried out using two-way ANOVA with Tukey's test. (**d**) The expression of CD3ɛ and TCRγδ in IL-17A-producing cells (EGFP^+^ cells of *Il17a*^*gfp*/*gfp*^ mice) in the repair tissue (*n*=3−8 per time point). (**e**) The number of γδ T cells and the ratio of IL-17A-producing γδ T cells in the repair tissue. Statistical analysis was carried out using one-way ANOVA with Dunnett's test. (**f**) Proliferation of γδ T cells after injury. BrdU^+^ γδ T cells were detected by flow cytometry (*n*=3 per time point). Statistical analysis was carried out using Student's *t*-test. Error bars denote the mean±s.e.m. *P<0.05 versus day 0; †*P*<0.05 at a time point.

The cellular source of IL-17A following bone injury was next explored. Immunohistological analysis of the repair tissue in IL-17A–GFP reporter mice (*Il17a*^*gfp/gfp*^ mice) showed that IL-17A^+^ cells were observed around the drill hole and the injured muscle, but they were hardly detected in the bone marrow (Fig. 5a,b and Supplementary Fig. 5a). The number of IL-17A^+^ cells significantly increased 2 and 7 days after injury and the density of these cells was higher in the drill hole than in the injured muscle at day 2 (Fig. 5c).

To characterize the IL-17A-producing cells after injury, we analysed the cells harvested from the repair tissue in *Il17a*^*gfp/gfp*^ mice. By flow cytometric analysis, we found that most of the IL-17A^+^ cells were CD3ɛ^+^TCRγδ^+^ γδ T cells (Fig. 5d and Supplementary Fig. 5b,c). The number of γδ T cells and the ratio of IL-17A^+^ cells increased in the repair tissue after injury (Fig. 5e and Supplementary Fig. 5d). The proliferation of γδ T cells in the repair tissue was remarkably increased 2 days after the injury (Fig. 5f). Although regulatory T cells were suggested to be involved in bone fracture healing^16^, Foxp3^+^ cells did not contribute to IL-17A production in this model as analysed in *Foxp3*^hCD2/hCD2^*Il17a*^*gfp*/*gfp*^ mice^17^ (Supplementary Fig. 5e).

To specifically examine the role of γδ T cells in bone regeneration, we analysed the bone regeneration in *Tcrd*^−/−^ mice, which are deficient in γδ T cells. Bone regeneration was significantly impaired in *Tcrd*^−/−^ mice (Supplementary Fig. 6a–d), indicating that γδ T cells play a crucial role in bone regeneration. In the injury tissue of *Tcrd*^−/−^ mice, *Il17a* expression was significantly lower than that of wild-type mice but not completely abrogated (Supplementary Fig. 6e). Analysis of surface markers of IL-17A-producing CD3ɛ^−^ cells by flow cytometry revealed that there was a Thy-1^+^ population in both wild-type and *Tcrd*^−/−^ mice, suggesting that type 3 innate lymphoid cells may compensate for the loss of IL-17A production by γδ T cells (Supplementary Fig. 6f).

Among the cytokines related to IL-17A, the expression of *Il1b* and *Il23p19*, which were reported to activate γδ T cells to produce IL-17A^18^,^19^, was robustly upregulated in the cells of the repair tissue in the early stage of bone regeneration, suggesting their contribution to IL-17A production (Supplementary Fig. 7).

**Figure 6:**
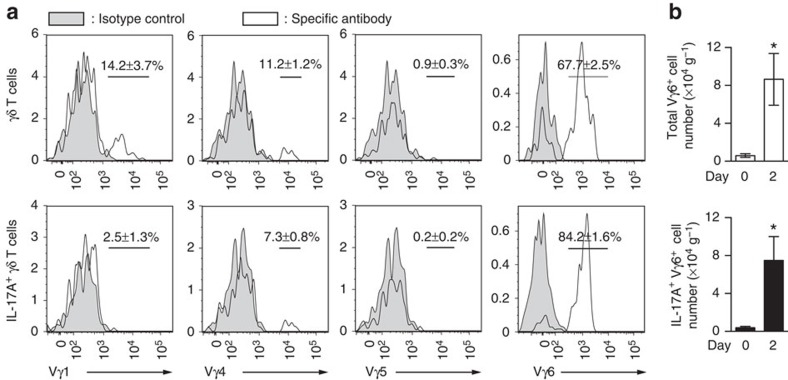
IL-17A-producing γδ T cells in the injury site are Vγ6^+^ cell subset. (**a**) The usage of Vγ chain of γδ T cells in the repair tissue after injury (*n*=3). 17D1 antibody recognizes Vγ5^+^ and Vγ6^+^ cells, but there were virtually no Vγ5^+^ γδ T cells observed, indicating IL-17A-producing γδ T cells in the injury site are enriched in the Vγ6^+^ cell subset. (**b**) The number of 17D1^+^ γδ T cells (upper) and IL-17A-producing 17D1^+^ γδ T cells (lower) in the repair tissue before and after injury (*n*=3 per time point). Statistical analysis was carried out using Student's *t* test. Error bars denote the mean±s.e.m. *P<0.05.

γδ T cells are divided into subsets expressing distinct TCR-Vγ chains, with each subset having its own characteristic tissue distribution and cytokine production pattern^20^,^21^. Approximately 70% of the γδ T cells in the repair tissue after the injury expressed Vγ6 (Fig. 6a)^22^. Furthermore, over 80% of IL-17A^+^ γδ T cells were Vγ6^+^ cells (Fig. 6a). Vγ6^+^ γδ T cells exhibited a remarkable increase in number 2 days after the injury (Fig. 6b). These results indicate that Vγ6^+^ γδ T cells play a key role in IL-17A production in musculoskeletal tissue, to promote bone regeneration after injury.

## Discussion

Although it has long been recognized that the immune system is closely related to bone fracture healing, the mechanisms linking them have been poorly understood. In this study, we found that IL-17A is highly induced after bone injury. Bone regeneration was impaired in *Il17a*^−/−^ mice, due to the decrease in osteoblastic bone formation. IL-17A promoted osteoblastogenesis of mesenchymal cells harvested from the repair tissue, in which PαS cells were enriched. We also found that Vγ6^+^ γδ T cells were the major source of IL-17A in bone regeneration process. Thus, our data demonstrate that Vγ6^+^ γδ T cells produce IL-17A to promote osteoblastic bone formation in bone regeneration.

γδ T cells are innate-like lymphocytes characterized by the expression of γδ TCRs. Unlike αβ T cells, these cells contain subsets bearing invariant TCRs. Invariant γδ TCRs are thought to recognize stress-induced self-antigens or damage-associated molecules, contributing to tissue surveillance^23^. Vγ6^+^ γδ T cells, which express the invariant Vγ6 chain, mainly reside in epithelial tissues and are reported to promote epithelial regeneration^11^. Our findings show that Vγ6^+^ γδ T cells reside in the musculoskeletal tissue of mesenchymal origin and are involved in bone regeneration, further defining γδ T cells as a maintainer of tissue homeostasis. The prompt immune responses through γδ T cells may be beneficial for initiating the rapid regeneration in tissue injury. γδ T cells can be stimulated directly by damage-associated molecules via the TCR or innate receptors such as Toll-like receptors in the injury site. γδ T cells produce IL-17A on stimulation by IL-1β and IL-23 (ref. 19), both of which were upregulated soon after injury (Supplementary Fig. 7). Activation of IL-17-producing γδ T cells by such stimuli would also constitute an effective response to bone fracture.

We found that IL-17A functions as an effector molecule derived from γδ T cells in bone regeneration. IL-17A, produced mainly by T_H_17 cells, has been characterized as an inflammatory cytokine that exerts diverse effects including the eradication of the pathogenic microorganisms and the induction of autoimmune inflammation^24^. IL-17A also plays an important role in the regulation of bone metabolism, but its function has been mainly studied in the context of bone resorption in autoimmune arthritis: IL-17A derived from T_H_17 cells in inflamed synovium stimulates receptor activator of nuclear factor-κB ligand (RANKL) expression on synovial fibroblasts, as well as local inflammation, resulting in exaggerated osteoclastic bone resorption^2^,^24^,^25^. However, in the present study, osteoclastic bone resorption in *Il17a*^−/−^ mice was not affected (Supplementary Fig. 2a–c). This is possibly because the number of IL-17A-producing cells in the repair tissue was very small in the late phase when osteoclastogenesis occurred (Fig. 5c). IL-17A-blocking antibody has been shown to be effective for chronic inflammatory diseases associated with excessive bone formation including spondyloarthropathy and psoriatic arthritis, suggesting the positive effect of IL-17A in bone formation^26^,^27^; however, the role of IL-17A on bone formation has been unclear.

IL-17A exerts distinct effects on bone formation depending on target cell types. In certain previous studies, IL-17A was shown to exert negative effects on the osteoblastogenesis in the culture of cells isolated from neonatal mouse calvaria^15^. They are thought to mainly contain mesenchymal cells of osteoblast lineage. On the other hand, IL-17A was shown to exert positive effects on immature mesenchymal cells including MSCs and myoblasts^28^,^29^. It is likely to be that IL-17A positively regulates the early stage of osteoblast proliferation and/or differentiation. It is reported that the osteogenic progenitors, not mature osteoblasts, are the major source of osteoblasts participating in bone regeneration^30^. Considering the results that high induction of IL-17A is limited to the short term after bone injury, it is likely to be that IL-17A plays a role in increasing the number of osteoprogenitor cells in the early phase of bone fracture healing, but the detailed mechanisms remain to be elucidated in future studies.

Binding of IL-17A with its receptor IL-17R results in the recruitment of Act1 and TNF receptor-associated factor (TRAF)2/4/5/6, which activates nuclear factor-κB, mitogen-activated protein kinases and C/EBPβ/δ (ref. 31). The specificity of the downstream signalling pathways is considered to be determined by the interaction of Act1 with different TRAF molecules. A recent study revealed that the TRAF4−ERK5 cascade is necessary for IL-17A-induced epidermal cell proliferation and is a dominant pathway in skin tumour due to the elevated expression of TRAF4 (ref. 32). These studies suggest that the balance of the expression of the signalling molecules downstream of IL-17R determines the cellular response.

We used the simple drill-hole injury model. Other fracture models with a large defect or treated with an unstable fixation involve endochondral bone formation, in which cartilage is formed within the bone defect and replaced by the bone. Although it remains unclear how IL-17A regulates such endochondral bone formation, it is possible that IL-17A promotes the healing process via proliferation of immature mesenchymal cells. As IL-17A is involved in bone and cartilage destruction in inflammation, careful future assessment of various fracture models is needed.

Thus, it is demonstrated that there is a crucial role for IL-17-producing γδ T cells in bone regeneration, suggesting that γδ T cells contribute to the maintenance of not only the epithelial barrier but also the supportive tissue underneath. γδ T-cell-based immunotherapy has long been studied in the treatment of cancer. Although it is necessary to develop the method to stimulate tissue-specific γδ T cells, this study suggests IL-17-producing γδ T cells to be an effective therapeutic target for bone fracture healing.

## Methods

### Animals and *in vivo* experiments

Mice were kept under specific pathogen-free conditions and all the experiments were performed with the approval of the Institutional Review Board at the University of Tokyo. C57BL/6 mice were purchased from Clea Japan, Inc. *Il17a*^−/−^ mice and *Il17f*^−/−^ mice were previously generated on a C57BL/6 background^33^,^34^. *Il17a*^*gfp/gfp*^ mice were obtained from BIOCYTOGEN. *Foxp3*^hCD2/hCD2^ mice were previously generated on a C57BL/6 background^17^. *Tcrd*^−/−^ mice were obtained from the Jackson Laboratory. For analyses on bone regeneration, female mice of 6–8 weeks old were used. For the analyses on inflammation, female and male mice of 6–8 weeks old were used.

For drill-hole injury, as described previously^13^, mice were anaesthetized with an intraperitoneal injection of pentobarbital sodium. Hair on the thigh was removed using an electric shaver and the skin underneath was disinfected with ethanol. An approximately 10-mm longitudinal skin incision was made immediately above and parallel to the femur. The bone surface was exposed by splitting the muscle mesiodistally and removing the periosteum. A drill hole with a diameter of 0.8–1.0 mm was made on the anterior portion of the diaphysis of the femur. The incised muscle and skin were closed with nylon sutures.

For the proliferation assay, mice were injected intraperitoneally with 150 μl of BrdU (Sigma) solution at the concentration of 10 mg ml^−1^ in saline (Otsuka Pharmaceutical Factory, Inc.), 1 day before killing.

For micro-CT analysis, mice were killed at specified time points and the femur was fixed with 70% ethanol until analysis. For the preparation of paraffin-embedded specimens, tissues were fixed by immersion in 4% paraformaldehyde. For the preparation of frozen specimens, tissues were fixed by perfusion and immersion, using the same fixative.

### Isolation of cells from mice

Tissues were obtained from the femur as depicted in Supplementary Fig. 1a. Bone marrow cells were harvested from the femur by introducing PBS into the bone marrow cavity. Erythrocytes were depleted by haemolysis using ammonium chloride (Sigma). To collect the repair tissue cells, the skin, skeletal muscles and periosteum were removed from the femur and the tissues in the drill hole, periosteum and skeletal muscle were digested in a collagenase (WAKO) solution at the concentration of 1 mg ml^−1^ dissolved in RPMI (Gibco). Debris was removed by filtration using 70 μm mesh (BD) and by density centrifugation using 40% Percoll (GE Healthcare) solution in RPMI, followed by haemolysis. Neonatal murine calvarial cells were isolated as described previously^35^: neonatal mice were killed by anaesthesia and disinfected by immersion in ethanol. Calvaria were dissected and digested in a digestion solution, in which 1 mg ml^−1^ of collagenase and 2 mg ml^−1^ of dispase were dissolved in α-MEM (Gibco). Debris was removed by filtration using 70 μm mesh and osteoblastic cells were collected.

### Quantitative reverse transcriptase–PCR

Total RNAs of the cells prepared as described above were extracted using TRIzol (Life Technologies) according to the manufacturer's instructions. First-strand complementary DNAs were synthesized using Superscript III reverse transcriptase (Invitrogen). Quantitative real-time reverse transcriptase–PCR was performed with a LightCycler (Roche) using SYBR Green (TOYOBO) according to the manufacturer's protocol. The expression level of mRNA was normalized with that of *Gapdh*. The primers for analysis of gene expression by real-time reverse transcriptase–PCR were as follows: for *Gapdh*, 5′-ACCCAGAAGACTGTGGATGG-3′ and 5′-CACATTGGGGGTAGGAACAC-3′; *Ifng*, 5′-GCGTCATTGAATCACACCTG-3′ and 5′-TGAGCTCATTGAATGCTTGG-3′; *Il4*, 5′-CCTCACAGCAACGAAGAACA-3′ and 5′-ATCGAAAAGCCCGAAAGAGT-3′; *Il17a*, 5′-TCCCTCTGTGATCTGGGAAG-3′ and 5′-AGCATCTTCTCGACCCTGAA-3′; *Il17f*, 5′-ATGGTGCTGTCTTCCTGACC-3′ and 5′-CAAAACCAGGGCATTTCTGT-3′; *Il22*, 5′-GCCCTGGACAGCTGTACTTCTA-3′ and 5′-ACACGAGTGCTACTTGGTTAGGA-3′; *Il6*, 5′-CCGGAGAGGAGACTTCACAG-3′ and 5′-CAGAATTGCCATTGCACAAC-3′; *Tnfa*, 5′-GCTGAGCTCAAACCCTGGTA-3′ and 5′-CGGACTCCGCAAAGTCTAAG-3′; *Runx2*, 5′-CCCAGCCACCTTTACCTACA-3′ and 5′-TATGGAGTGCTGCTGGTCTG-3′; *Sp7*, 5′-ACTGGCTAGGTGGTGGTCAG-3′ and 5′-GGTAGGGAGCTGGGTTAAGG-3′; *Col1a1*, 5′-GAGCGGAGAGTACTGGATCG-3′ and 5′-GTTAGGGCTGATGTACCAGT-3′; *Alpl*, 5′-AACCCAGACACAAGCATTCC-3′ and 5′-GCCTTTGAGGTTTTTGGTCA-3′; *Il1b*, 5′-CAGGCAGGCAGTATCACTCA-3′ and 5′-TGTCCTCATCCTGGAAGGTC-3′ and *Il23p19*, 5′-AATAATGTGCCCCGTATCCA-3′ and 5′-AGGCTCCCGTTTGAAGATGT-3′.

### Micro-CT analysis

CT scanning was performed using ScanXmate-D090S105 and Xsys software (Comscantecno). Three-dimensional microstructural image data were reconstructed by coneCT express software (White Rabbit) and the structural indices were calculated using TRI/3D Bon software (Ratoc System Engineering). A sequence of images within the bone defect was chosen for analyses. Region of interest (ROI) was set as the cylindrical region bordered by the defect edge with an axis of ∼800 μm and a thickness of 100 μm.

### Histological and histomorphometric analyses

For haematoxylin-eosin (HE) staining, tartrate-resistant acid phosphatase (TRAP) staining and the immunofluorescence for BrdU, tissues fixed by 4% paraformaldehyde underwent decalcification in OSTEOSOFT (Merck Millipore) at 4 °C for 3 weeks and were then embedded in paraffin after dehydration. Six-micrometre-thick sections were made. Sectioning of the paraffin-embedded samples was performed along the longitudinal axis of the femur. HE staining was carried out by staining with haematoxylin (Muto Pure Chemicals) for 3 min followed by 2 min of staining with eosin (Wako). TRAP staining was performed at room temperature for ∼20 min followed by nuclear counterstaining with haematoxylin. The immunofluorescence for BrdU was performed using BrdU Immunohistochemistry Kit (Abcam) in combination with streptavidin conjugated with fluorescence dye (Life Technologies) and nuclear counter staining by Hoechst33342. Bone formation and bone resorption were quantified using Win ROOF software (Mitani Corporation). BrdU^+^ cells were counted using Tissue Quest software (Novel Science).

For the immunofluorescence for green fluorescent protein (GFP), non-decalcified frozen sections of 5 μm thick were made as previously reported (Kawamoto's method)^36^, with minor modifications: tissues fixed in 4% paraformaldehyde were freeze embedded with carboxymethyl cellulose in a cooled hexane. An adhesive film was put on the cut surface of the samples. Sections were made with a tungsten carbide blade. Carboxymethyl cellulose compound was washed away from the section before staining. The sections were incubated with primary antibody solution containing a rabbit anti-GFP polyclonal antibody (Abcam) at 4 °C for 1 h, followed by incubation with a secondary antibody solution containing goat anti-rabbit IgG antibody conjugated with fluorescence dye (Life Technologies) for 1 h. Nuclei were stained with Hoechst33342.

### *In vitro* differentiation of osteoblasts

Osteoblastogenesis of murine calvarial cells was induced as previously described^35^: calvarial cells were resuspended in α-MEM containing 10% fetal bovine serum and seeded in a culture dish (10 cm dish for one mouse). After incubation for 2 days, cells were resuspended in an osteogenic medium (50 μg ml^−1^ ascorbic acid, 10 nM dexamethasone and 10 mM β-glycerophosphate) and seeded in 24-well plates (1.0 × 10^4^ cells per well). The culture medium was changed every third day. Recombinant mouse BMP-2 (Osteogenetics GmbH) and IL-17A (Peprotech) were added coincidentally with the induction of osteoblastogenesis and the change of the media at a final concentration of 500 and 50 ng ml^−1^, respectively.

Injury-associated mesenchymal cells were obtained by depleting CD45^+^ cells from the repair tissue cells of wild-type mice on day 3 with autoMACS Pro Separator (Miltenyi Biotec) using rat anti-mouse CD45 antibody conjugated with PE (eBioscience) and anti-PE Micro Beads (Miltenyi Biotec). Dead cells were depleted after being cultured in a dish for 1 day. Non-haematopoietic adherent cells (1.0 × 10^4^ cells per well) in the osteogenic medium were seeded in 24-well plates. The culture medium was changed every third day. BMP-2 and IL-17A were added coincidentally with the induction of osteoblastogenesis and the change of the media.

PαS cells were sorted out by using FACS Aria III (BD Biosciences). Cells (3.0 × 10^3^) per well in the osteogenic medium supplemented with BMP-2 and IL-17A were seeded in 48-well plates, at a final concentration of 500 and 10 ng ml^−1^, respectively.

ALP staining was performed as previously described^35^. Cultured cells were fixed with 4% paraformaldehyde for 15 min on ice. After rinsing with PBS, the cells were stained for 15 min with ALP staining solution (Napthol AS-MX phosphatase, 0.06 mg ml^–1^; *N*,*N*-dimethylformamide, 1%; and Fast blue BB salt, 1 mg ml^–1^ in 0.1 M Tris-HCl pH 8.0). The staining solution was washed away with tap water. Stained cells were air dried. ALP activity measurement was performed as follows. Cells were collected in a lysis buffer (12 mM Tris-HCl pH 8.0, 12 mM MgCl_2_ and 0.05% Triton X in dH_2_O). Cells were subjected to three freeze–thaw cycles, ultrasonic disruption and homogenization. The debris was spun down and the supernatant was collected. ALP activity was measured using LabAssay ALP (Wako Pure Chemical Industries) and the total protein was measured using DC Protein Assay (Bio-Rad), according to the manufacturers' instructions. ALP activity was normalized to total protein.

Alizarin Red S staining and the measurement of mineralization were performed as previously described^37^ on day 14 (injury-associated mesenchymal cells) or 21 (calvarial cells). Cells were fixed as described above. After rinsing with dH_2_O, the staining was performed with Alizarin Red S staining solution (0.02 g ml^–1^ Alizarin Red S in dH_2_O pH 4.2). The staining solution was washed away with dH_2_O. Stained cells were air dried. Bone nodules in the culture of calvarial cells was counted after Alizarin Red S staining was performed on day 21 (ref. 35). For the measurement of mineralization, Alizarin Red S was extracted with 10% acetic acid and assessed for absorbance at 405 nm.

An *in vitro* BrdU incorporation assay was performed using Cell Proliferation ELISA, BrdU (chemiluminescent) (Roche Applied Science) according to the manufacturer's instructions.

### Flow cytometry

Cells were maintained at 4 °C throughout the procedure. Dead cells were distinguished as the cells that incorporated 2.5 ng ml^−1^ of Ethidium Monoazide Bromide (Life Technologies) and nonspecific binding was blocked with anti-CD16/32 (93, Biolegend) at a dilution of 1/100. The antibodies used for flow cytometric analysis were as follows. Anti-mouse CD3ɛ (145-2C11, eBioscience), CD11b (M1/70, Biolegend and eBioscience), CD29 (HM81-1, Biolegend), CD31 (390, eBioscience), CD34 (RAM34, eBioscience), CD44 (IM7, Biolegend), CD45 (30-F11, Biolegend and eBioscience), CD90.2 (30-H12, eBioscience), CD140α (APA5, eBioscience), CD217 (PAJ-17R, eBioscience), Sca-1 (D7, eBioscience), TCRγδ (GL3, eBioscience), TCR-Vγ1 (2.11, Biolegend), TCR-Vγ4 (UC3-10A6, Biolegend), TCR-Vγ5 (536, Biolegend), TER-119 (TER-119, eBioscience) and anti-human CD2 (RPA-2.10, eBioscience) were used at a dilution of 1/250. Anti-rat IgM (MRM-47, Biolegend) and streptavidin conjugated with fluorescence dyes (Biolegend, eBioscience and BD Pharmingen) were used at a dilution of 1/200. Monoclonal antibody 17D1 specific for TCR-Vγ6Vδ1 and TCR-Vγ5Vδ1 was kindly provided by Dr Tigelaar (Yale University). The filtered culture supernatant of 17D1 hybridoma cells was directly added to each well. For the *in vivo* proliferation assay, BrdU Flow Kit (BD Pharmingen) was used. Data were acquired on a FACSCanto II (BD Biosciences) and analysed using FlowJo software (TREE STAR).

### RNA sequencing

Total RNA of cultured PαS cells was extracted using Maxwell 16 LEV simplyRNA Cells and Tissue Kit (Promega). cDNA was synthesized and amplified using SMART-Seq v4 Ultra Low Input RNA Kit for Sequencing (Clontech Laboratories). Data were acquired on an Ion Proton (Thermo Fisher) and analysed using CLC Genomics Workbench (CLC).

### Statistical analysis

*P*-values were calculated using Student's *t*-test or analysis of variance (ANOVA) with Dunnett's (for one-way ANOVA) or Tukey's (for two-way ANOVA) multiple-comparison test. Differences with a *P*-value of <0.05 were considered significant (*P<0.05; †*P*<0.05; NS, not significant, throughout the paper). All the data are expressed as mean±s.e.m.

## Additional information

**Accession codes:** The RNA-Seq data have been deposited in GEO under the accession code GSE77172.

**How to cite this article:** Ono, T. *et al*. IL-17-producing γδ T cells enhance bone regeneration. *Nat. Commun.* 7:10928 doi: 10.1038/ncomms10928 (2016).

## Supplementary Material

Supplementary Information Supplementary Figures 1-7.
