# Supplementary material for: IL-17-producing γδ T cells enhance bone regeneration
Source: Nat Commun. 2016 Mar 11;7:10928. doi: 10.1038/ncomms10928 (PMC4792964; doi:10.1038/ncomms10928)
Supplement: Supplementary Information — Supplementary Figures 1-7. [file ncomms10928-s1.pdf]

Supplementary Figure 1

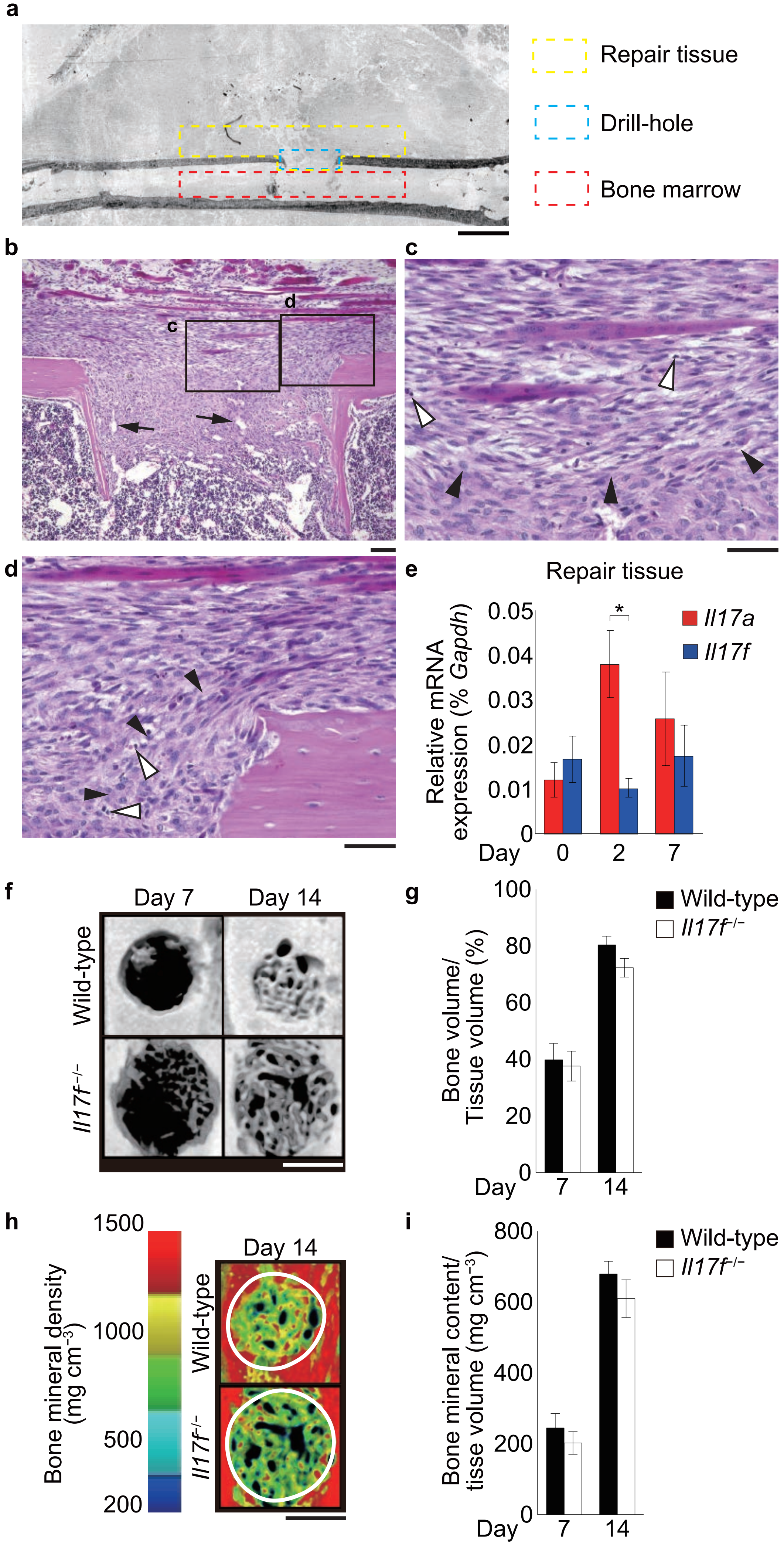

Supplementary Figure 1. IL-17F does not contribute to bone regeneration. **(a)** Typical bright field microscopic image of the injured femur that shows the experimental system. Tissues of the boxed areas were harvested and the cells isolated from these tissues were analyzed in the following experiments. Scale bar, 1 mm. **(b)** HE staining of the injury site in the wild-type mice. The arrows indicate the vasculature invading into the injury site. Scale bar, 100 μm. **(c)** The junction of the regenerating tissue in the bone defect and regenerating muscle fibers. Scale bar, 50 μm. **(d)** The junction of the regenerating tissue and thickening periosteum. The arrowheads point out the merging junction of the tissue in the bone defect and the neighboring tissues. The white arrowheads indicate the infiltrated immune cells. Scale bar, 50 μm. **(e)** Comparison of the mRNA expression of *Il17a* and *Il17f* in the repair tissue of wild-type mice (n = 3−6 per time point per genotype). Expression levels were normalized to *Gapdh*. **(f)** Micro-CT images of the drill-holes in the wild-type and *Il17f*$^{-/-}$ mice after injury. Scale bar, 500 μm. **(g)** Quantification of bone formation in the drill-holes (n = 4−8 per time point per genotype). **(h)** Bone mineral density was visualized by coloring the micro-CT images according to the CT values. The lines denote the original shapes of the drill-holes. **(i)** Bone mineral content in the drill-holes (n = 4−8 per time point per genotype). Statistical analysis was carried out using Student's t test at each time point throughout the Supplementary Figure 1. Error bars denote the mean ± s.e.m. *$P<0.05$.

Supplementary Figure 1 continued.

Supplementary Figure 2

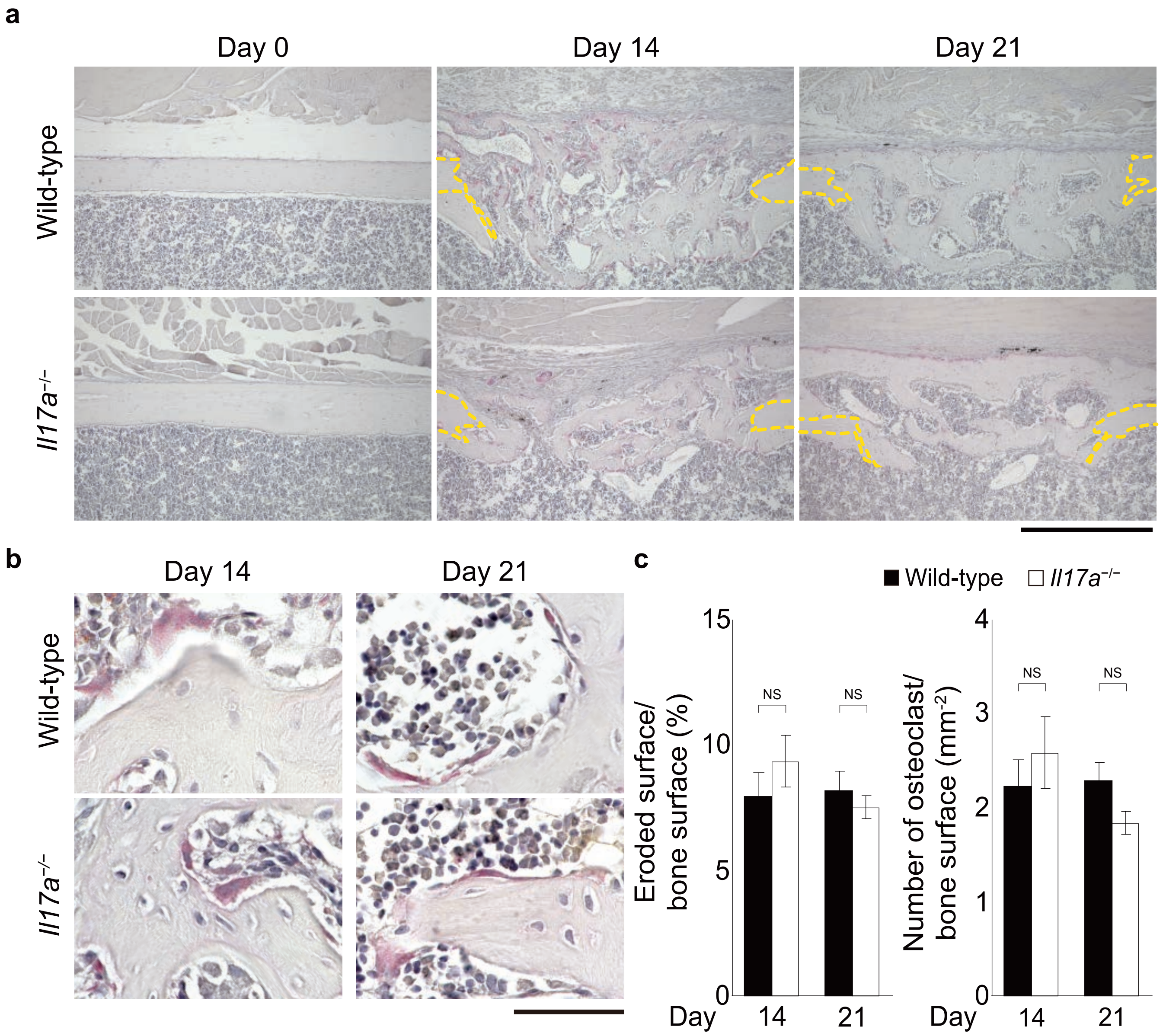

Supplementary Figure 2. IL-17A does not affect osteoclastic bone resorption after bone injury. (a) Osteoclasts of the femur of the wild-type and *Il17a*$^{-/-}$ mice were stained with TRAP staining. The lines in the images represent the edge of the drill-hole. Scale bar, 500 μm. **(b)** High magnification images of the newly formed bone covered with TRAP^{+}osteoclasts. Scale bar, 50 μm. **(c)** The eroded surface and the number of osteoclasts in the regenerating tissue were quantitated by bone histomorphometric analyses (n = 3 per time point per genotype, 2−6 representative sections per each femur). Statistical analysis was carried out using Student's t test at each time point. Error bars denote the mean ± s.e.m. NS, not significant.

Supplementary Figure 3

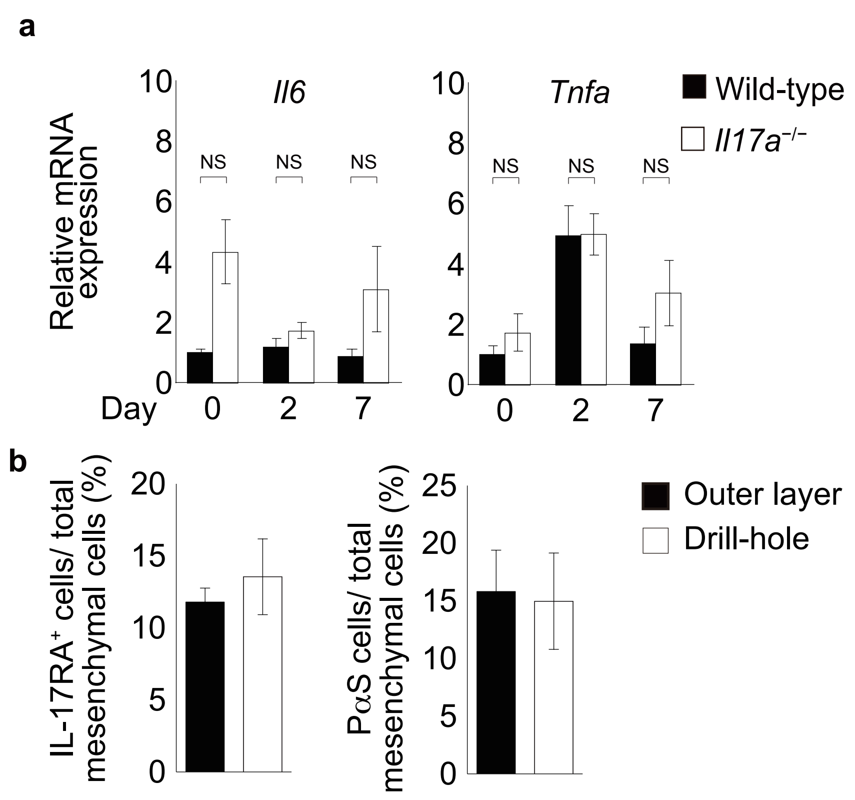

Supplementary Figure 3. IL-17A promotes bone formation independently of IL-6 and TNF. (a) mRNA expression of *Il6* and *Tnfa* in the repair tissues of wild-type and *Il17a*$^{-/-}$ mice (n = 3 per time point per genotype). **(b)** The ratio of IL-17RA^{+} cells and the ratio of PαS cells to total mesenchymal cells, which were CD45^{-}Ter119^{-}CD11b^{-}CD31^{-}CD34^{-}. Cells were obtained from the repair tissue in the drill-hole and the outer layer of the repair tissue of the same femur (n = 3). Statistical analysis was carried out using Student's t test at each time point. Error bars denote the mean ± s.e.m. NS, not significant.

Supplementary Figure 4

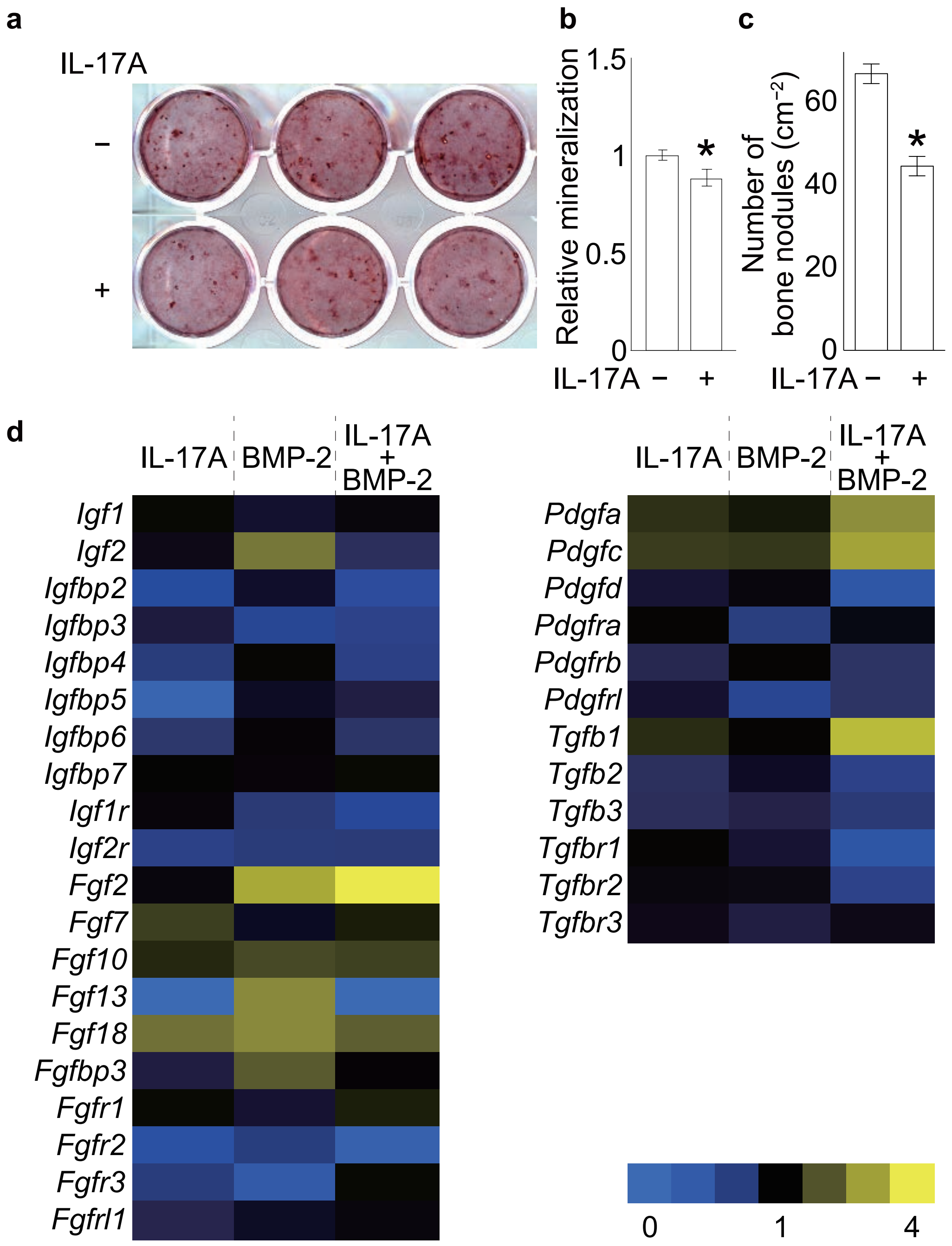

Supplementary Figure 4. IL-17A differently regulates the osteoblastogenesis of mesenchymal cells from different origins. **(a)** The effect of IL-17A on the *in vitro* mineralization of murine neonatal calvarial cells analyzed by Alizarin Red S staining. **(b)** Mineralization was quantified as the amount of Alizarin Red S in each well. **(c)** The number of bone nodules formed in each well was counted. Data was obtained from 3 independent experiments with triplicate wells. Statistical analysis was carried out using Student's *t* test. Error bars denote the mean ± s.e.m. *P<0.05. **(d)** mRNA expression profile of PαS cells after the culture in the presence of IL-17A and/or BMP-2. Cells were harvested at day 7. The colored bar shows the fold upregulation of gene expression in comparison to the cells cultured in the absence of IL-17A and BMP-2.

Supplementary Figure 5

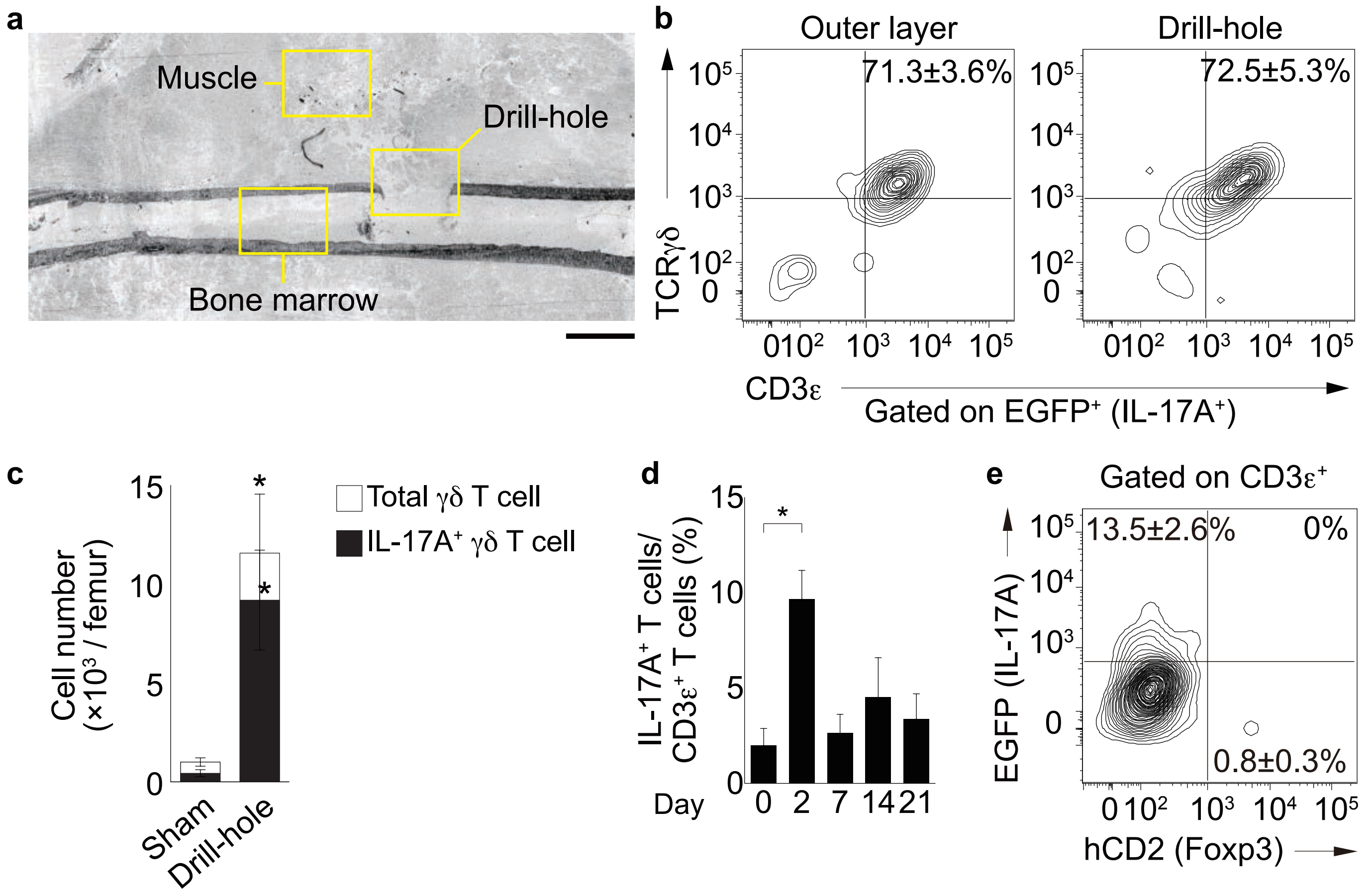

Supplementary Figure 5. γδ T cells in the repair tissue are the major source of IL-17A after bone injury. **(a)** Typical bright field microscopic image of the injured femur. Boxed areas were analyzed in **Figures 5a-c**. Scale bar, 1 mm. **(b)** The ratio of γδ T cells to IL-17A-producing cells. Cells were obtained from the repair tissue in the drill-hole and the outer layer of the repair tissue of the same femur (n = 3). **(c)** The number of total γδ T cells and the number of IL-17A-producing γδ T cells in the repair tissue of sham-operated and drilled femur (n = 3). Statistical analysis was carried out using Student's t test. **(d)** The ratio of IL-17A-producing cells to total T cells (n = 3−8 per time point). Statistical analysis was carried out using one-way ANOVA with Dunnett's test. **(e)** The contribution of Foxp3^{+} cells to IL-17A production. The expression of Foxp3 was detected as the expression of hCD2 using *Foxp3*$^{\mathrm{hCD2/hCD2}}$*Il17a*$^{gfp/gfp}$ mice (n = 3). Error bars denote the mean ± s.e.m. *P<0.05.

Supplementary Figure 6

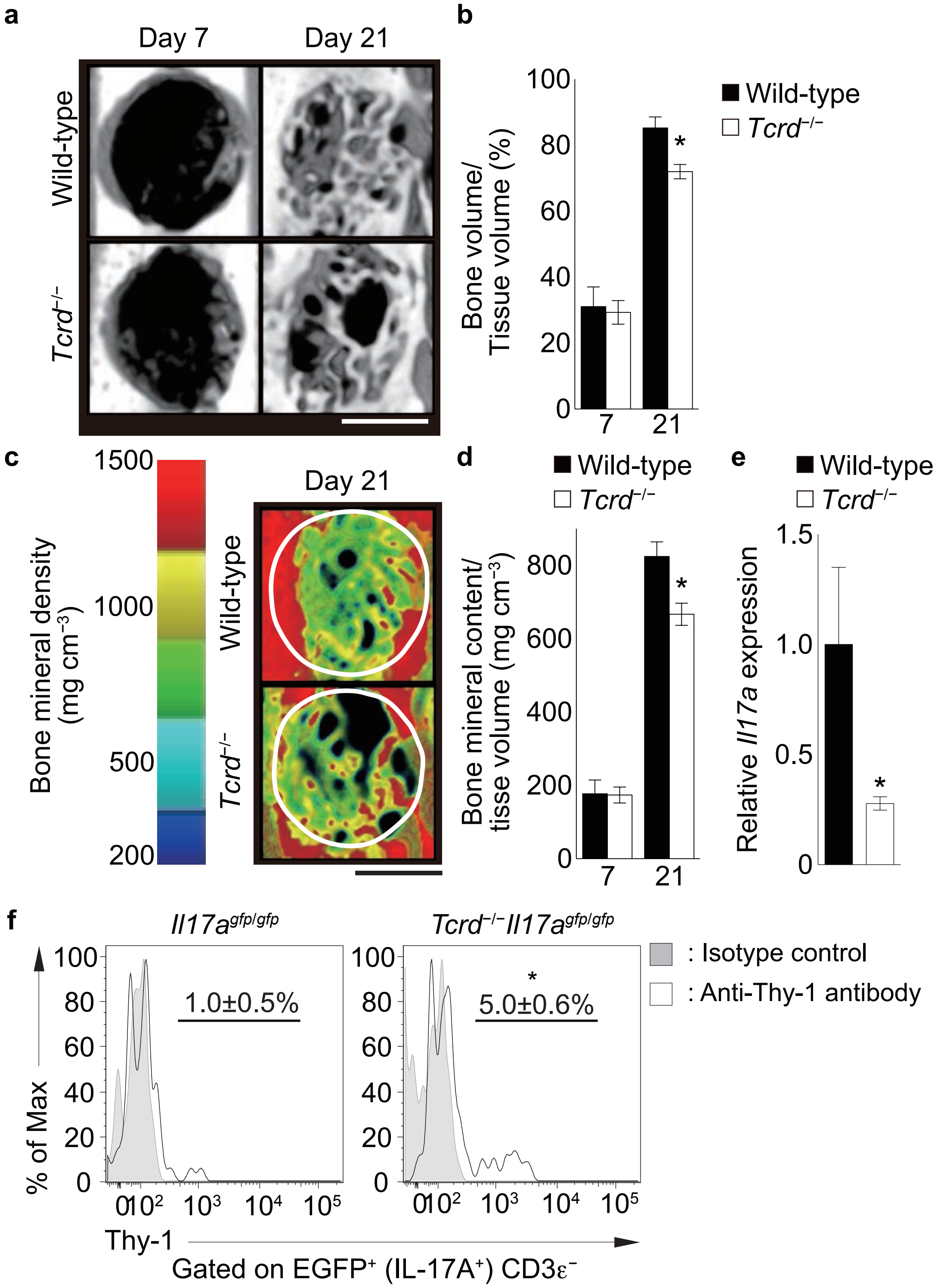

Supplementary Figure 6. γδ T cells contribute to bone regeneration. **(a)** Micro-CT images of the drill-holes in the wild-type and *Tcrd*$^{-/-}$ mice after injury. Scale bar, 500 μm. **(b)** Quantification of bone formation in the drill-holes (*n* = 4–5 per time point per genotype). **(c)** Visualization of the bone mineral density of the newly formed bone in the drill-holes. Images were constructed by coloring the micro-CT images according to the CT values. The lines denote the original shapes of the drill-holes. Scale bar, 500 μm. **(d)** Bone mineral content in the drill-holes (*n* = 4–5 per time point per genotype). **(e)** *Il17a* mRNA expression in the injury tissue of the wild-type and *Tcrd*$^{-/-}$ mice at day 2 (*n* = 3 per genotype). **(f)** Thy-1 expression on IL-17A-producing CD3ε^{-} cells in the wild-type and *Tcrd*$^{-/-}$ mice in the *Il17a*$^{gfp/gfp}$ background. (*n* = 3 per genotype). Statistical analysis was carried out using Student's *t* test for each time point in the Supplementary Figure 6. Error bars denote the mean ± s.e.m. *P<0.05.

Supplementary Figure 7

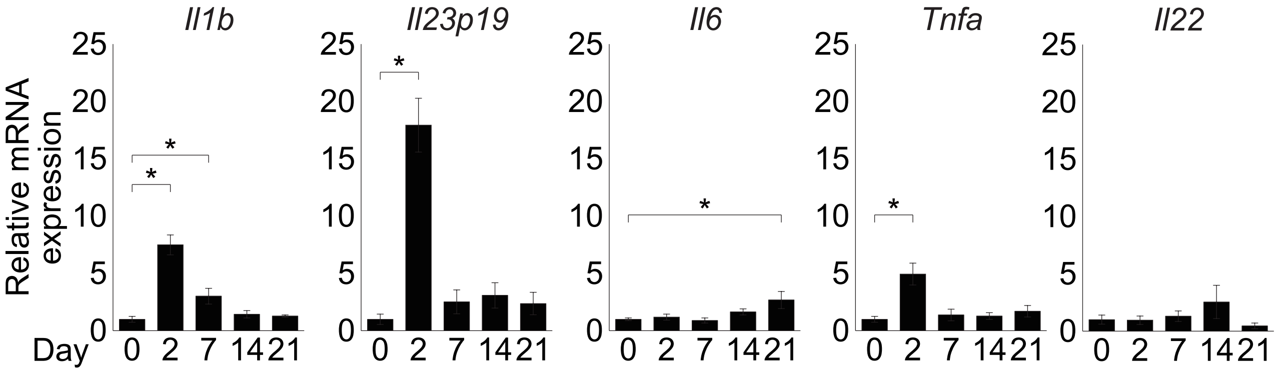

Supplementary Figure 7. IL-1β and IL-23 are robustly upregulated in the repair tissue after bone injury. mRNA expression of *Il1b*, *Il23p19*, *Il6*, *Tnfa* and *Il22* in the repair tissue of wild-type mice (*n* = 3 −8 per time point). Statistical analysis was carried out using one-way ANOVA with Dunnett's test. Error bars denote the mean ± s.e.m. *P<0.05.
